# Sigma-1 Receptor Stimulation Rescues FTD/ALS Mutant TDP43-Induced Disruption of the VAPB-PTPIP51 ER–Mitochondria Tethering Proteins via Inhibition of GSK3β

**DOI:** 10.3390/cells15171536

**Published:** 2026-08-26

**Authors:** Kerry Blair, Philippe Gosset, Raquel Martinez-Serra, Gábor M. Mórotz, Sandra M. Martín-Guerrero, Patricia Gomez-Suaga, Joseph Atherton, Jacqueline C. Mitchell, Wendy Noble, Christopher C. J. Miller, Andrea Markovinovic

**Affiliations:** 1 Department of Basic and Clinical Neuroscience, Institute of Psychiatry, Psychology and Neuroscience, King’s College London, London WC2R 2LS, UKpatricia.gomez-suaga@kcl.ac.uk (P.G.-S.); chris.miller@kcl.ac.uk (C.C.J.M.); 2 Randall Centre for Cell and Molecular Biophysics, King’s College London, London WC2R 2LS, UK; 3 Department of Pharmacology and Pharmacotherapy, Semmelweis University, H-1089 Budapest, Hungary; 4 Center for Pharmacology and Drug Research & Development, Semmelweis University, H-1085 Budapest, Hungary; 5 Consejo Superior de Investigaciones Científicas (CSIC), Instituto de Parasitología y Biomedicina López-Neyra, 18016 Granada, Spain; 6 Departamento de Bioquímica y Biología Molecular y Genética, Facultad de Enfermería y Terapia Ocupacional, Universidad de Extremadura, 10071 Cáceres, Spain; 7 Centro de Investigación Biomédica en Red en Enfermedades Neurodegenerativas-Instituto de Salud Carlos III (CIBER-CIBERNED-ISCIII), 28029 Madrid, Spain; 8 Instituto Universitario de Investigación Biosanitaria de Extremadura (INUBE), 10071 Cáceres, Spain; 9 Department of Clinical and Biomedical Sciences, University of Exeter, Exeter EX4 4QJ, UK; 10 Department of Anatomy, Faculty of Medicine, University of Rijeka, 51000 Rijeka, Croatia

**Keywords:** VAPB, PTPIP51, Sigma-1 receptor, TDP43, amyotrophic lateral sclerosis, frontotemporal dementia, PRE-084, ANAVEX2-73

## Abstract

Signalling between the ER and mitochondria regulates a number of key cellular functions that are damaged in frontotemporal dementia and related amyotrophic lateral sclerosis (FTD/ALS). This signalling involves close physical contacts between the two organelles that are mediated by the VAPB-PTPIP51 ER–mitochondria “tethering” proteins. A number of studies have shown that mutant genes which cause familial FTD/ALS disrupt the VAPB-PTPIP51 tethers and that this involves activation of GSK3β. TDP43 is one such mutant and altered TDP43 metabolism is central to FTD/ALS pathogenesis. Loss of Sigma-1 receptor function is also seen in FTD/ALS and there is evidence that Sigma-1 receptor agonists can repair damaged ER–mitochondria signalling. However, the underlying mechanisms are not properly understood. In this study, we show that the reference Sigma-1 receptor agonist PRE-084 stimulates VAPB-PTPIP51 binding and rescues FTD/ALS mutant TDP43-induced disruption to the VAPB-PTPIP51 interaction and linked ER–mitochondria Ca^2+^ delivery. We also show that these effects involve inhibition of the kinase GSK3β, a known negative regulator of VAPB-PTPIP51 binding. Finally, we show that ANAVEX2-73, a further Sigma-1 receptor agonist which is in clinical trials for Alzheimer’s disease, also stimulates VAPB-PTPIP51 binding via GSK3β inhibition. Our findings provide novel insights into the mechanisms by which Sigma-1 receptor agonists influence defective ER–mitochondria signalling in FTD/ALS.

## 1. Introduction

The Sigma-1 receptor is a protein located in the endoplasmic reticulum (ER) that regulates a number of physiological functions, including Ca^2+^ signalling, mitochondrial bioenergetics, lipid metabolism, autophagy and ER stress responses (see reviews [1,2,3,4,5]). Also, there is evidence that Sigma-1 receptor stimulation can have beneficial effects for several nervous system disorders including substance addiction, schizophrenia, depression and some neurodegenerative diseases [1,3,4]. Despite these findings, endogenous Sigma-1 receptor ligands are still not properly characterised, although a number of synthetic compounds are known to stimulate its activity [6].

Within the ER, the Sigma-1 receptor locates to a specialised region that forms close contacts with mitochondria; these ER regions are termed mitochondria-associated ER membranes (MAMs) (see reviews [1,2,3,4]). MAMs facilitate cross-talk and signalling between ER and mitochondria, and this enables the two organelles to respond to physiological signals in an orchestrated manner (see reviews [7,8,9,10,11]). A primary function of communication between the ER and mitochondria is to enable the transfer of Ca^2+^ from ER stores to mitochondria through inositol 1,4,5-trisphosphate (IP3) receptor-dependent mechanisms. This process involves the release of Ca^2+^ from the ER via IP3 receptors, followed by its transfer into mitochondria through channels including the voltage-dependent anion channel (VDAC) and the mitochondrial Ca^2+^ uniporter (MCU). Mitochondrial Ca^2+^ uptake is essential for supporting ATP production through the tricarboxylic acid cycle, and in this way, ER–mitochondria contacts and signalling regulate cellular bioenergetics (see reviews [7,8,9,10,11]). A well-characterised function of the Sigma-1 receptor is to facilitate IP3 receptor delivery of Ca^2+^ to mitochondria, and there is evidence that this involves stimulating ER–mitochondria contacts [12,13,14,15].

The processes underlying the formation of close contacts between the ER and mitochondria are not properly understood; however, it is widely accepted that this involves specialised “tethering” proteins that bring ER membranes into proximity with mitochondria, thereby facilitating communication between the two organelles. A number of tethers have been described, and one of the best characterised involves an interaction between the integral ER protein vesicle-associated membrane protein-associated protein-B (VAPB) and the outer mitochondrial membrane protein, protein tyrosine phosphatase interacting protein 51 (PTPIP51) (also known as regulator of microtubule dynamics-3 and family with sequence similarity 82 member A2) [16,17,18]. VAPB-PTPIP51-mediated tethering modulates a range of important ER–mitochondria signalling functions, including IP3 receptor-mediated delivery of Ca^2+^ to mitochondria, lipid synthesis and metabolism, autophagy, as well as synaptic activity in neurons [7,16,19,20,21,22,23,24].

Autosomal recessive mutations in *SIGMAR1* (the gene encoding the Sigma-1 receptor) cause some familial forms of frontotemporal dementia (FTD), amyotrophic lateral sclerosis (ALS) and related motor neuropathies; these mutations include frameshift and single amino acid changes [13,15,25,26,27,28,29]. FTD represents the second most common type of presenile dementia after Alzheimer’s disease, while ALS is the most prevalent form of motor neuron disease [30]. Increasing evidence indicates that FTD and ALS are closely linked at the clinical, pathological and genetic levels. Thus, FTD and ALS patients can display features of both diseases, mutations in the same genes cause familial forms of FTD and ALS, and both diseases display similar pathologies [30]. Notably, accumulations of TAR DNA binding protein-43 (TDP43) in affected neurons are a hallmark pathology of FTD and ALS, and mutations in *TARDBP* (which encodes TDP43) are linked to genetic forms of both diseases [30,31,32,33,34,35,36,37,38,39]. Such findings support the notion that damage to TDP43 metabolism is a key feature in many forms of FTD/ALS [30,38,40]. Interestingly, the FTD/ALS mutant TDP43 is now known to disrupt ER–mitochondria signalling and linked IP3 receptor delivery of Ca^2+^ to mitochondria, and this disruption involves breaking of the VAPB-PTPIP51 tethers [16,41]. Moreover, damage to the VAPB-PTPIP51 tethers by TDP43 involves activation of glycogen kinase-3β (GSK3β); GSK3β is thus a negative regulator of VAPB-PTPIP51 binding and ER–mitochondria signalling [16,42,43,44,45,46,47,48].

FTD/ALS-linked *SIGMA1R* mutations are known to be loss-of-function mutations and indeed, genetic ablation of *SIGMA1R* causes some ALS-type locomotor deficits, axonal degeneration and motor neuron loss in mice [13,14,15,49,50]. Moreover, FTD/ALS *SIGMA1R* mutants and Sigma-1 receptor loss disrupt both ER–mitochondria contacts and IP3 receptor delivery of Ca^2+^ to mitochondria [13,14]. Such findings argue that Sigma-1 receptor stimulation with small-molecule agonists such as PRE-084 and ANAVEX2-73 will have therapeutic benefits for FTD/ALS and other neurodegenerative diseases, and there is evidence to support this notion [13,14,51,52,53,54]. However, the mechanisms by which such agonists enhance ER–mitochondria contact formation and associated signalling are not properly understood. In this study, we show that the reference Sigma-1 receptor agonist PRE-084 stimulates VAPB-PTPIP51 binding and rescues FTD/ALS mutant TDP43-induced disruption to the VAPB-PTPIP51 interaction and linked ER–mitochondria Ca^2+^ delivery. We also show that these effects involve, at least in part, inhibition of GSK3β. Finally, we show that ANAVEX2-73, a further Sigma-1 receptor agonist which has been linked to several neurodegenerative diseases and is in clinical trials for Alzheimer’s disease, also stimulates VAPB-PTPIP51 binding via inhibition of GSK3β. Together, our findings provide novel insights into the mechanisms by which drugs that stimulate the Sigma-1 receptor influence defective ER–mitochondria contacts and signalling in FTD/ALS.

## 2. Materials and Methods

### 2.1. Plasmids, Antibodies and Other Reagents

Plasmids encoding EGFP-tagged TDP43-Q331K in pEGFP-C1, as well as VAPB and PTPIP51 NanoBiT constructs, have been reported previously [16,41]. The rabbit anti-VAPB antibody (3504) and rat anti-PTPIP51 antibody (skr9) were used as described previously, with dilutions of 1:200 for proximity ligation assay (PLA) and 1:500 for immunoblot analysis [17]. Chicken anti-microtubule-associated protein 2 (MAP2) antibody (Genetex) was applied for immunostaining at a dilution ratio of 1:500. Detection of serine-9-phosphorylated GSK3β was performed using a rabbit antibody from Cell Signalling at 1:100. For immunoblotting, the following antibodies were used at a dilution ratio of 1:500: rabbit anti-IP3 receptor type 1 (Synaptic Systems), mouse anti-VDAC1 (Abcam), rabbit anti-MCU (Atlas), rabbit anti-protein disulfide isomerase (PDI; Proteintech), rabbit anti-TOM20 (Proteintech), and mouse anti-b-tubulin (Sigma). Species-specific secondary antibodies (goat or donkey anti-mouse, anti-rabbit, and anti-chicken IgGs) conjugated to Alexa Fluor 488, 555, 594, or 647 were obtained from Invitrogen and Jackson ImmunoResearch Laboratories. PRE-084, BD1047 and NE100 were obtained from Tocris; ANAVEX2-73 was obtained from Selleckchem.

### 2.2. Cell Culture and Transfection

SH-SY5Y cells were sourced from the European Collection of Cell Cultures and maintained in Dulbecco’s modified Eagle’s medium/F12 (1:1) supplemented with 3.15 g/L of glucose (Gibco). The culture medium was further enriched with 10% (*v*/*v*) foetal bovine serum, GlutaMAX, 100 IU/mL of penicillin, and 100 µg/mL of streptomycin. Plasmid transfections were performed using Lipofectamine 2000 (Invitrogen) at a ratio of 1 µg of DNA to 2 µL of reagent, following the manufacturer’s protocol. Primary cortical neurons were isolated from embryonic day 18 rat embryos and seeded onto poly-D-lysine-coated glass coverslips (Marienfeld). Cells were maintained in Neurobasal medium supplemented with B27, GlutaMAX, 100 IU/mL of penicillin, and 100 µg/mL of streptomycin (Gibco). Transfection of neurons was carried out at 7 days in vitro (DIV7) using Lipofectamine 2000 under the same conditions described above, and analyses were conducted 24 h post-transfection.

### 2.3. SDS-PAGE and Immunoblotting

Protein samples for SDS-PAGE and subsequent immunoblot analysis were prepared in accordance with previously published methods [55]. Cells were first washed with phosphate-buffered saline (PBS) and then collected by scraping into ice-cold RIPA buffer composed of 150 mM of NaCl, 1% Triton X-100, 0.5% sodium deoxycholate, and 0.1% SDS in 50 mM of Tris-HCl (pH 8.0), supplemented with protease inhibitors (Complete, Roche). Following lysis, samples were mixed with SDS sample buffer and denatured by heating at 96 °C for 5 min. Proteins were resolved by electrophoresis on 10% polyacrylamide gels using a Mini-PROTEAN 2 system (Bio-Rad) under discontinuous buffer conditions. After separation, proteins were transferred onto nitrocellulose membranes with a 0.2 µm pore size (BioTrace NT, VWR, Lutterworth, UK) using a Mini Trans-Blot transfer apparatus (Bio-Rad) for 1.5 h. Membranes were blocked for 1 h in Tris-buffered saline (TBS) containing 5% (*w*/*v*) non-fat milk and 0.1% (*v*/*v*) Tween-20. Incubation with primary antibodies was carried out in the same blocking solution supplemented with Tween-20, followed by washing in TBS/Tween-20. Membranes were then incubated with IRDye-labelled secondary antibodies, and protein bands were detected and quantified using an Odyssey CLx near-infrared imaging system (Li-Cor Biosciences, Cambridge, UK).

### 2.4. Calcium Measurements

Mitochondrial Ca^2+^ levels were assessed using the fluorescent indicator Rhod-2 AM (Invitrogen) as previously reported [16,17,23,42,56,57]. Fluorescence data were processed using Nikon NIS-Elements AR 6, and mitochondrial Ca^2+^ levels were quantified as the change in fluorescence intensity following oxotremorine-M stimulation relative to the mean baseline signal prior to treatment (F/F0).

### 2.5. Proximity Ligation Assays and Immunostaining

Protein–protein interactions between VAPB and PTPIP51 were examined using proximity ligation assays (PLA) performed with the Duolink In Situ Orange kit (Sigma), following previously reported protocols and the manufacturer’s guidelines [24,41,44,55]. After completion of the signal amplification steps, neuronal samples were co-immunostained for MAP2 to verify neuronal identity; MAP2 staining was performed on all neuronal samples and only MAP2-positive cells were analysed. Detection of serine-9-phosphorylated GSK3β was performed as reported previously [41]. Consistent with earlier reports, only cells exhibiting relatively low levels of transfected proteins (as determined by fluorescence intensity) were included in the analysis to minimise potential artefacts associated with overexpression [41,58,59,60,61,62,63].

### 2.6. NanoBiT Assays

NanoBiT assays were carried out using SH-SY5Y cells seeded in white, opaque 96-well plates at a density of 3 × 10^4^ cells per well. Measurements were performed in live cells in accordance with the manufacturer’s protocol (Promega). Cells were transfected with 100 ng of NanoBiT plasmid DNA as described above, and after 24 h, they were treated with either vehicle control (DMSO) or the PRE-084 or ANAVEX2-73 Sigma-1 receptor agonist for 1 h. Control experiments included treatment with 10 µM of PRE-084 or 10 µM of ANAVEX2-73 in combination with either Sigma-1 receptor antagonist BD1047 (10 µM) or NE-100 (10 µM). Following treatment, the culture medium was replaced with 100 μL of phenol red-free Opti-MEM (Gibco). Luminescence was initiated by adding 25 μL of Nano-Glo Live Cell assay reagent to each well. Signals were recorded using a GloMax Navigator luminometer over a 15 min period, with an integration time of 0.5 s per well.

### 2.7. GSK3β ELISAs

Quantification of total and serine-9-phosphorylated GSK3β levels was carried out using the GSK3β (Total/Phospho) InstantOne™ ELISA kit (Thermo Fisher Scientific), following the manufacturer’s protocol. Signal detection was performed using a CLARIOstar multi-mode plate reader (BMG LabTech).

### 2.8. Statistical Analyses

Data analysis was conducted using Microsoft Excel (Microsoft Corporation) and GraphPad Prism (version 9.3.1; GraphPad Software Inc.). Specific statistical details are provided in the corresponding figure legends. In some figures, the common term not significant (ns) is used; this means the *p* value is greater than 0.05.

## 3. Results

### 3.1. The Sigma-1 Receptor Agonist PRE-084 Promotes VAPB-PTPIP51 Binding in Cultured Neuronal Cells

To begin to understand the mechanisms by which Sigma-1 receptor stimulation enhances ER–mitochondria contacts and signalling, we monitored the effect of the reference Sigma-1 receptor agonist PRE-084 on VAPB-PTPIP51 binding. PRE-084 has been used in numerous studies that investigated the therapeutic potential of Sigma-1 receptor stimulation in FTD/ALS [13,14,52,53,64,65,66,67].

To investigate the effect of PRE-084 on VAPB-PTPIP51 interactions, we used a NanoLuc Binary Technology (NanoBiT) luciferase assay. This approach is based on fusing the small (SmBiT) and large (LgBiT) subunits of NanoLuc luciferase to the cytosolic N- and C-termini of VAPB and PTPIP51 respectively. Following transfection of these constructs into cultured cells, protein–protein interactions bring the luciferase fragments into close proximity, enabling reconstitution of an active enzyme and generation of a luminescent signal proportional to the interaction strength. The NanoBiT vectors involve the weak HSV-TK promoter which generates low-level expression of fusion proteins. These assays have been described and validated in our previous work [41]. For these experiments, we used neuronal SH-SY5Y cells. This clonal cell line has been utilised in many studies of the VAPB-PTPIP51 tethers, and so their use here permits direct comparisons with data from previous work [41,44,46,56,57].

**Figure 1:**
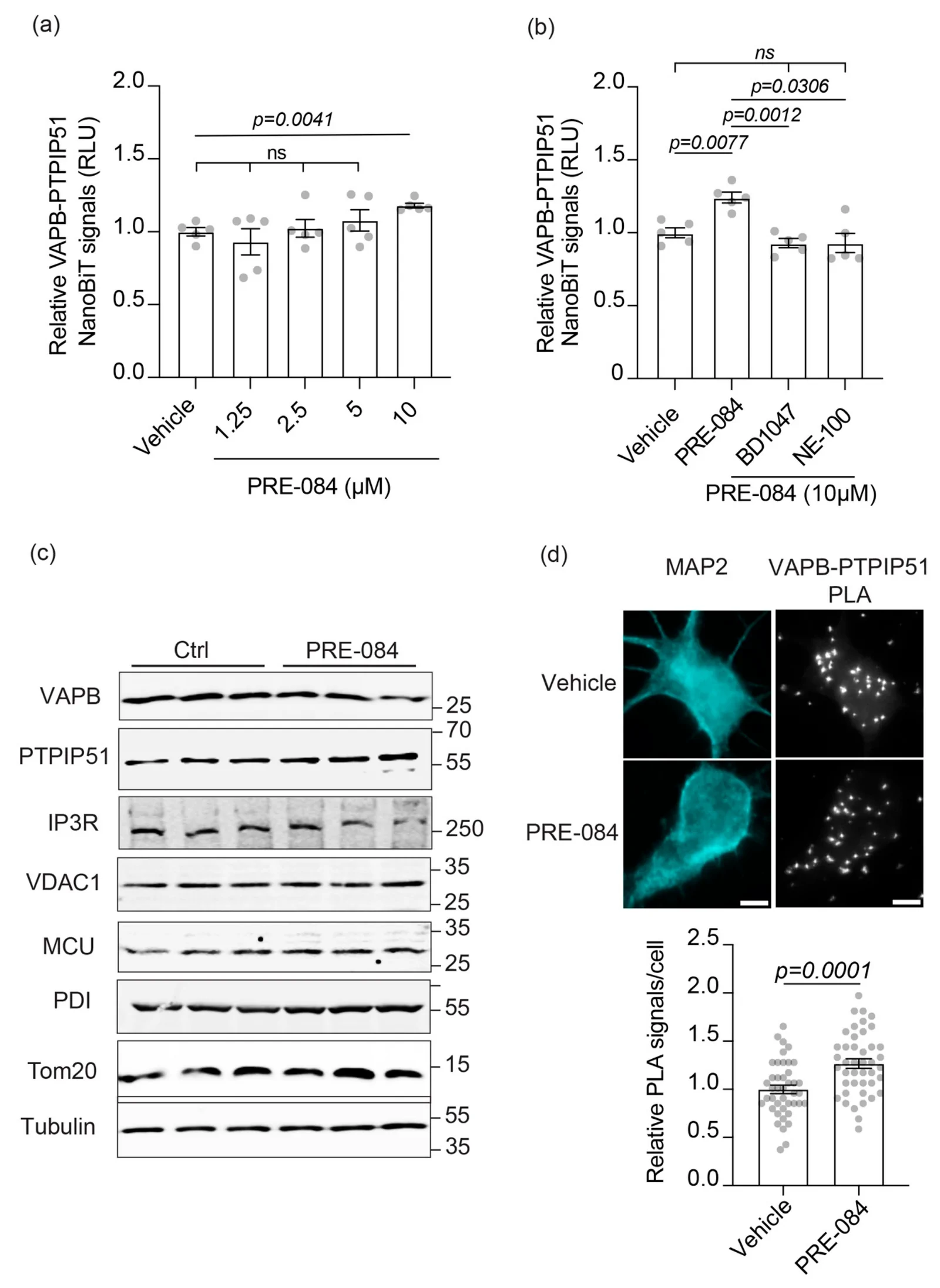
PRE-084 stimulates VAPB-PTPIP51 binding. (**a**) The effect of PRE-084 on VAPB-PTPIP51 binding was determined in SH-SY5Y cells using NanoBiT luciferase assays. Cells were co-transfected with SBiT-VAPB and PTPIP51-LBiT plasmids and subsequently treated with vehicle or increasing concentrations of PRE-084 for 1 h. Data were obtained from 5 independent experiments (N = 6 per experiment). Error bars are SEM. An amount of 10 µM of PRE-084 induced a significant increase in VAPB-PTPIP51 binding. Statistical analysis was carried out using Welch’s one-way ANOVA and Dunnett’s T3 multiple comparisons test. *p* value is shown; ns, not significant. W(4, 9.184) = 7.383. (**b**) Treatment with the Sigma-1 receptor antagonists BD1047 and NE-100 block the stimulatory effect of PRE-084 on VAPB-PTPIP51 binding. SH-SY5Y cells were transfected with VAPB+PTPIP51 NanoBiT plasmids and then treated with 10 µM of PRE-084 and either vehicle, 10 µM of BD1047 or 10 µM of NE-100 for 1 h. Data were obtained from 5 independent experiments (N = 5 per experiment). Error bars are SEM. Statistical analysis was carried out using Welch’s one-way ANOVA and Dunnett’s T3 multiple comparisons test. *p* values are shown; ns, not significant. W(3, 8.721) = 13.07. (**c**) PRE-084 does not affect expression of VAPB, PTPIP51 or other ER–mitochondria signalling-related proteins. SH-SY5Y cells were treated with vehicle or 10 µM of PRE-084 for 1 h and samples were analysed by SDS-PAGE and immunoblotting; molecular mass markers are shown on the right. Immunoblot signals were quantified and normalised to β-tubulin signals. No differences in the levels of VAPB, PTPIP51, IP3 receptor type 1 (IP3R), VDAC1, MCU, PDI, or Tom20 were detected. Statistical analyses were performed using two-tailed Welch’s *t*-test (N = 3 for all proteins). VAPB t(2.284) = 2.448, *p* = 0.1185; PTPIP51 t(2.852) = 3.186, *p* = 0.0535; IP3R t(2.801) = 1.345, *p* = 0.2771; VDAC1 t(3.38) = 0.8706, *p* = 0.4414; MCU t(2.432) = 1.077, *p* = 0.3767; PDI t(2.614) = 3.352, *p* = 0.0539; Tom20 t(2.883) = 1.088, *p* = 0.3591. (**d**) PRE-084 stimulates VAPB-PTPIP51 binding in PLAs in cortical neurons. Representative images are shown for neurons treated with either vehicle or 10 µM of PRE-084 for 1 h. Neuronal identity was confirmed by MAP2 immunostaining (shown in cyan). Scale bar = 5 µm. Data were obtained from 42–43 cells from 3 independent experiments. Error bars are SEM. Statistical analysis was performed using 2-tailed Welch’s *t*-test; *p* = 0.0001. t(82.28) = 4.032.

We monitored VAPB-PTPIP51 in the NanoBiT assays following treatment of the cells with vehicle or increasing concentrations of PRE-084. Treatment with 10 µM of PRE-084 significantly enhanced VAPB-PTPIP51 binding in these assays (Figure 1a). This finding is in line with many previous studies that show 10 µM of PRE-084 induces effective responses in other Sigma-1 receptor-linked functional assays (e.g., [68,69,70]). Whilst PRE-084 is a highly selective Sigma-1 receptor agonist that has been used in many studies, including ones on FTD/ALS [13,14,52,53,64,65,66,67], we confirmed that this effect of PRE-084 on VAPB-PTPIP51 binding involved Sigma-1 receptor stimulation by treating the cells with PRE-084 in combination with the Sigma-1 receptor antagonist BD1047 or NE-100. Co-treatment with BD1047 or NE-100 abolished the stimulatory effect of PRE-084 on VAPB-PTPIP51 binding (Figure 1b).

To determine whether the increased binding of VAPB to PTPIP51 following PRE-084 treatment involved changes in the levels of either protein, we monitored their expression on immunoblots. We also monitored the expression of IP3 receptor-1, VDAC1 and MCU, which mediate VAPB-PTPI51-regulated delivery of Ca^2+^ to mitochondria, Tom20 and PDI as markers for mitochondria and ER, respectively, and tubulin as a loading control. PRE-084 treatment did not affect the expression of any of these proteins (Figure 1c).

To complement these experiments, we also quantified the effect of PRE-084 treatment on VAPB-PTPIP51 binding using proximity ligation assays (PLAs) in DIV 7 cultured rat cortical neurons. Cortical neurons are affected in FTD. We and others have utilised such PLAs to quantify changes in VAPB-PTPIP51 binding in many related studies [16,24,41,42,44,46]. As was the case in the NanoBiT assays in SH-SY5Y cells, treatment with 10 µM of PRE-084 induced a significant increase in VAPB-PTPIP51 binding in these neurons (Figure 1d). Thus, PRE-084 stimulation of the Sigma-1 receptor enhances binding of both transfected and endogenous VAPB-PTPIP51 in two different cellular assays.

### 3.2. PRE-084 Rescues Disrupted VAPB-PTPIP51 Binding and Linked IP3 Receptor Delivery of Ca^2+^ to Mitochondria Induced by FTD/ALS Mutant TDP43-Q331K

**Figure 2:**
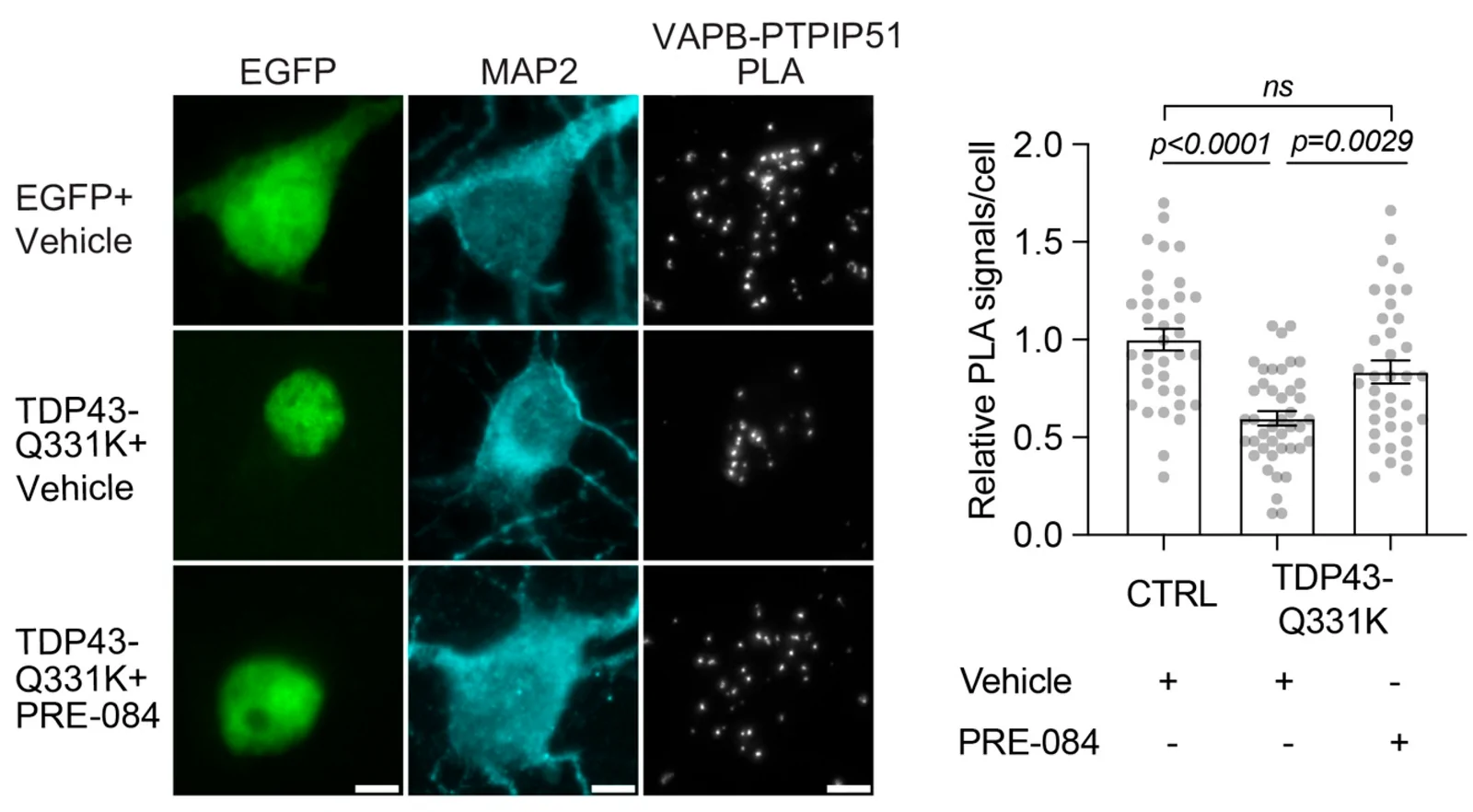
PRE-084 rescues FTD/ALS mutant TDP43-induced disruption to VAPB-PTPIP51 binding in cortical neurons. Representative images show VAPB-PTPIP51 PLA signals in neurons expressing either EGFP control or EGFP-TDP43-Q331K following treatment with either vehicle or 10 µM of PRE-084 for 1 h. TDP43-Q331K expression was identified via the EGFP signal, and neuronal identity was verified by MAP2 immunostaining (shown in cyan). Scale bar = 5 μm. Data were obtained from 36–42 cells from 3 independent experiments. Error bars are SEM. Statistical analysis was performed using one-way ANOVA and Tukey’s post hoc test. *p* values are shown; ns, not significant. F(2, 112) = 16.54.

Several familial FTD/ALS-linked mutants of TDP43, including TDP43-Q331K, have been shown to disrupt VAPB-PTPIP51 binding and linked ER–mitochondria signalling [16,41]. To determine whether Sigma-1 receptor stimulation can rescue these defects, we transfected rat cortical neurons with control EGFP or EGFP-TDP43-Q331K plasmid, treated the cells with PRE-084 or vehicle, and monitored VAPB-PTPIP51 binding using PLAs. These studies revealed that TDP43-Q331K reduced VAPB-PTPIP51 binding and that this effect was rescued by PRE-084 (Figure 2).

**Figure 3:**
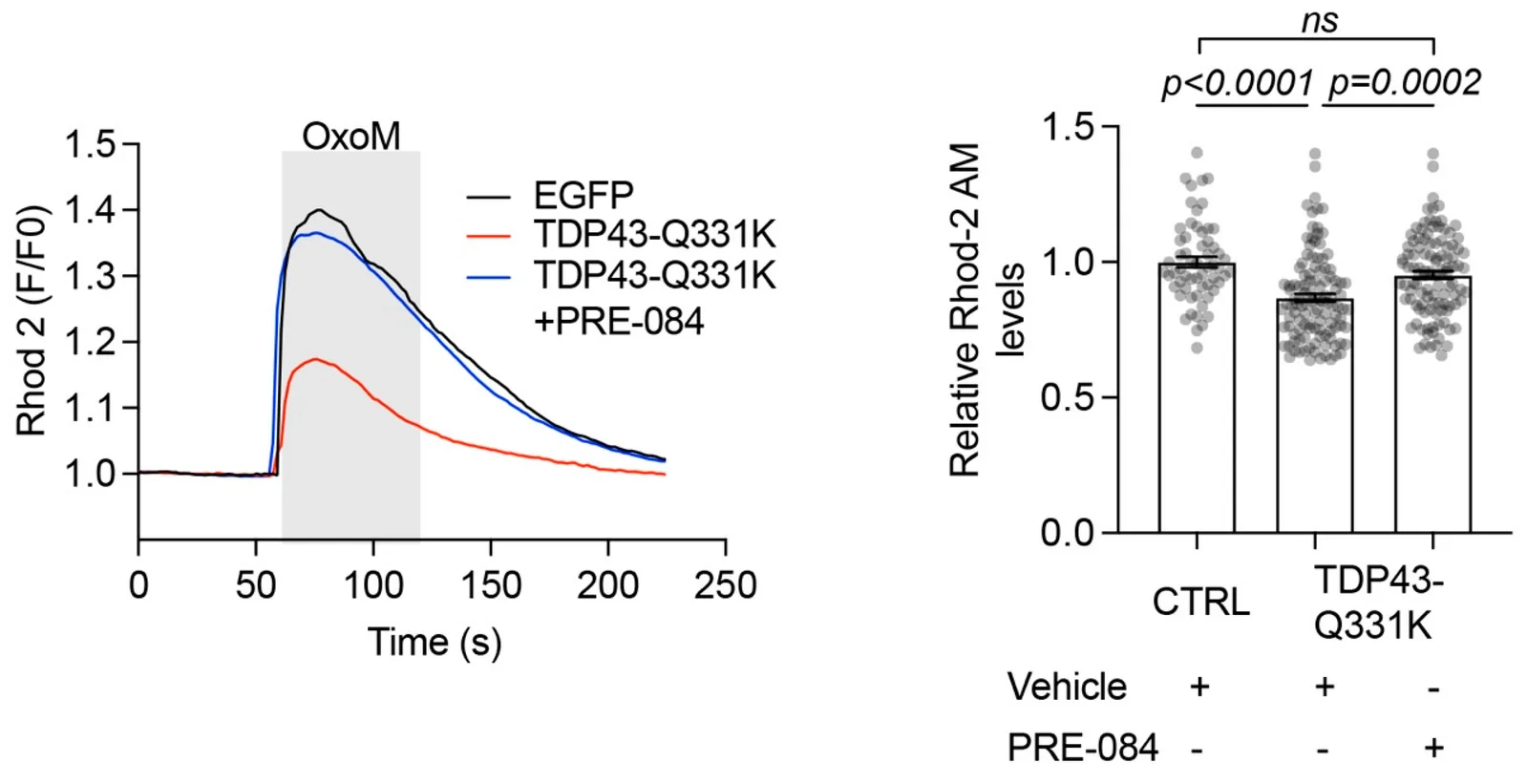
PRE-084 rescues IP3 receptor delivery of Ca^2+^ to mitochondria impaired by FTD/ALS mutant TDP43. SH-SY5Y cells expressing either EGFP control or EGFP-TDP43-Q331K plasmids were treated with vehicle or 10 µM of PRE-084 for 1 h as indicated. Mitochondrial Ca^2+^ levels were then measured using the fluorescent indicator Rhod-2 AM. Ca^2+^ release was triggered by application of oxotremorine-M (OxoM). Representative Rhod-2 fluorescence traces are shown on the left, with the period of OxoM stimulation indicated by the shaded region. Normalised peak responses are presented in the bar graph on the right. Expression of TDP43-Q331K reduced mitochondrial Ca^2+^ uptake, and this effect was blocked by PRE-084 treatment. Data were obtained from 60–115 cells from 3 independent experiments. Error bars are SEM. Statistical analysis was performed using one-way ANOVA and Tukey’s post hoc test. *p* values are shown; ns, not significant. F(2, 281) = 16.62.

A primary function of the VAPB-PTPIP51 tethers is to mediate IP3 receptor delivery of Ca^2+^ to mitochondria, and mutant TDP43 has been shown to impair this Ca^2+^ transfer [16,41,42,44,46,57]. To examine whether PRE-084 influences this process, we assessed mitochondrial Ca^2+^ uptake in both control cells and those expressing TDP43-Q331K. These experiments were carried out in SH-SY5Y cells, a well-established neuronal model frequently used to study ER–mitochondria Ca^2+^ signalling in the context of neurodegenerative diseases [41,44,46,56,57]. This use of SH-SY5Y cells is therefore advantageous as it enables better comparisons with earlier studies. In line with previous studies [16,41], expression of TDP43-Q331K reduced mitochondrial Ca^2+^ uptake following IP3 receptor stimulation. However, treatment with PRE-084 completely abrogated this effect (Figure 3). Thus, Sigma-1 receptor stimulation with PRE-084 rescues TDP43-Q331K-induced disruption to VAPB-PTPIP51 binding and associated IP3 receptor delivery of Ca^2+^ to mitochondria.

### 3.3. PRE-084 Inhibits GSK3β Activity and Rescues TDP43-Q331K-Induced Activation of GSK3β

**Figure 4:**
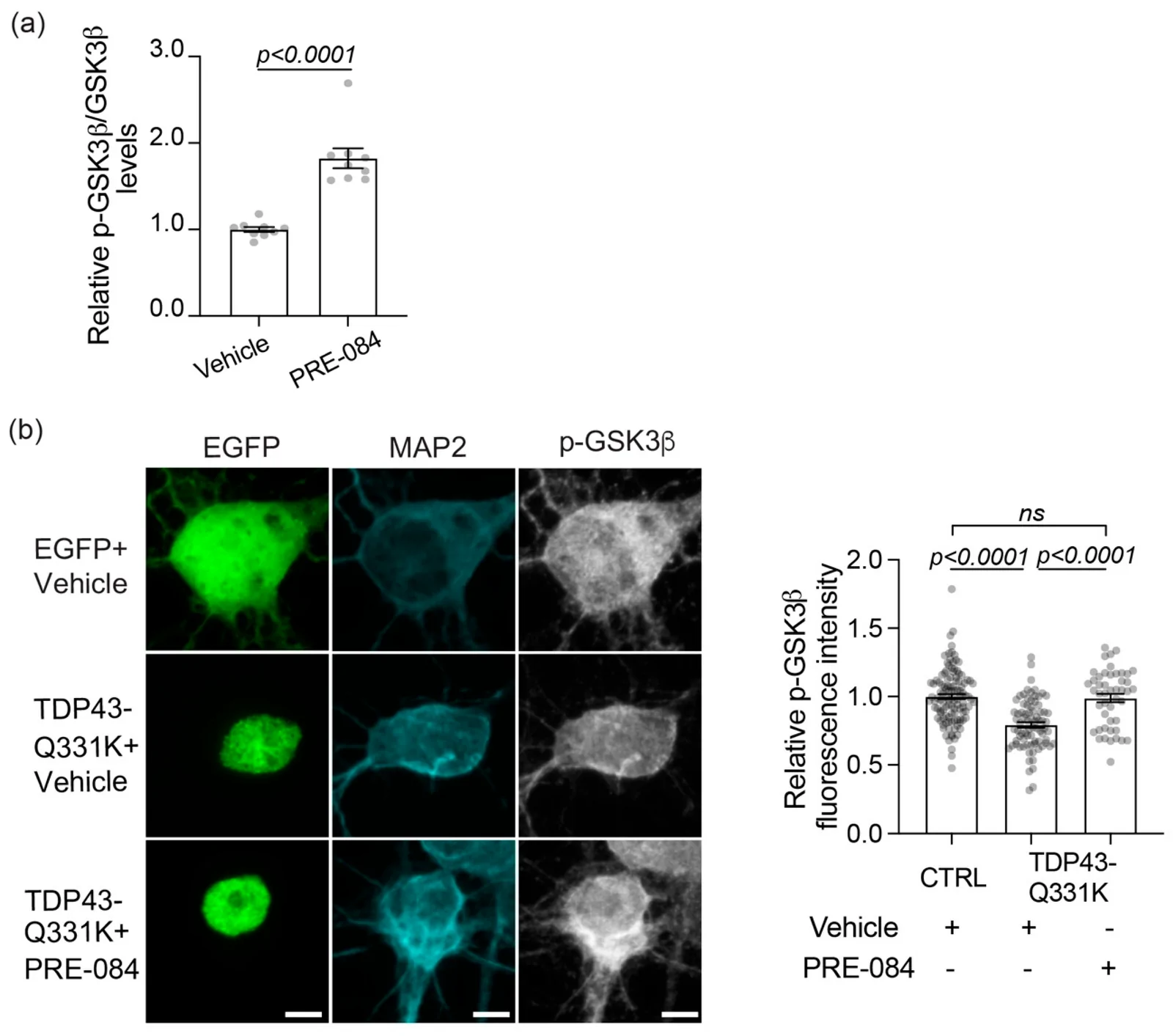
PRE-084 reduces GSK3β activity in SH-SY5Y cells and rescues mutant TDP43-induced GSK3β activation in cortical neurons. (**a**) The effect of PRE-084 (10 µM for 1 h) on GSK3β activity was determined using ELISAs to quantify the levels of total and serine-9-phosphorylated GSK3β. The levels of serine-9-phosphorylated GSK3β (p-GSK3β) are shown after normalisation to total GSK3β levels and are displayed as relative to vehicle-treated control cells. Data were obtained from 9 independent experiments. Error bars are SEM. Statistical analysis was performed using Welch’s *t*-test. *p* value is shown. t(9.058) = 6.898. (**b**) Representative images of cortical neurons expressing EGFP control or EGFP-TDP43-Q331K following treatment with vehicle or PRE-084 as indicated. Cells were immunostained for serine-9-phosphorylated GSK3β and MAP2 (shown in cyan) to confirm neuronal identity. Scale bar = 5 μm. Quantification of mean fluorescence intensity is shown in the accompanying bar chart after normalisation to EGFP + vehicle-treated control cells (CTRL). Data were obtained from 47–114 cells from 3 independent experiments. Error bars are SEM. Statistical analysis was performed using one-way ANOVA with Tukey’s post hoc test. *p* values are shown; ns, not significant. F(2, 237) = 28.76.

Several studies have shown that the toxicity associated with mutant TDP43 is at least partly mediated through increased GSK3β activity [16,41,43,45,47,48]. Regulation of GSK3β is strongly influenced by phosphorylation at serine-9, a modification that inhibits its kinase activity [71]. Importantly, FTD/ALS-linked mutant TDP43, FUS, C9orf72 and Tau have all been shown to impair VAPB–PTPIP51 tethering and associated IP3 receptor-dependent transfer of Ca^2+^ to mitochondria through a mechanism involving reduced serine-9 phosphorylation and consequent activation of GSK3β [7,16,19,41,42,44,46,72,73]. We therefore investigated whether PRE-084 could inhibit GSK3β activity by altering serine-9 phosphorylation. To do so, we first used ELISAs to quantify the relative levels of serine-9-phosphorylated GSK3β in SH-SY5Y cells treated with vehicle or PRE-084. PRE-084 increased serine-9 GSK3β phosphorylation in these assays (Figure 4a).

We next examined whether PRE-084 might inhibit TDP43-Q331K-induced activation of GSK3β in rat cortical neurons. To do so, we quantified serine-9-phosphorylated GSK3β levels in EGFP-TDP43-Q331K-expressing neurons following PRE-084 treatment. We have utilised this approach in previous studies that involved analyses of the effects of ursodeoxycholic acid on VAPB-PTPIP51 binding and GSK3β activity [41]. Compared to control EGFP-transfected cells, transfection of TDP43-Q331K induced a significant decrease in GSK3β serine-9 phosphorylation, which is in line with findings from previous studies [16,41]. However, treatment with PRE-084 alleviated this effect (Figure 4b). Together, these findings demonstrate that activation of the Sigma-1 receptor by PRE-084 rescues TDP43 induced disruption to VAPB-PTPIP51 binding via inhibition of GSK3β, a known mediator of TDP43 toxicity.

### 3.4. ANAVEX2-73, a Sigma-1 Receptor Agonist in Clinical Trials for Neurodegenerative Diseases, Stimulates VAPB-PTPIP51 Binding and Inhibits GSK3β Activity

PRE-084 is the most intensively studied and best characterised Sigma-1 receptor agonist and shows therapeutic potential in a number of experimental models of FTD/ALS [13,52,53,64,65,66,67]. Despite these findings, PRE-084 has not made it to the clinic as a treatment for FTD/ALS or other human neurodegenerative diseases. ANAVEX2-73 is a further Sigma-1 receptor agonist that displays therapeutic potential in some Alzheimer’s disease models and in more recent Phase2/3 clinical trials for Alzheimer’s disease [6,74,75,76,77,78]. However, unlike PRE-084, ANAVEX2-73 does not act solely as a Sigma-1 receptor agonist but also binds to the muscarinic acetylcholine M1 and ionotropic N-methyl-D-aspartate receptors, albeit with lower affinity [6]. Thus, experimental studies to dissect FTD/ALS disease mechanisms cannot be solely attributed to Sigma-1 receptor stimulation using ANAVEX2-73. Nevertheless, the potential clinical benefit of ANAVEX2-73 in dementia led us to examine its effect on the VAPB-PTPIP51 tethers.

**Figure 5:**
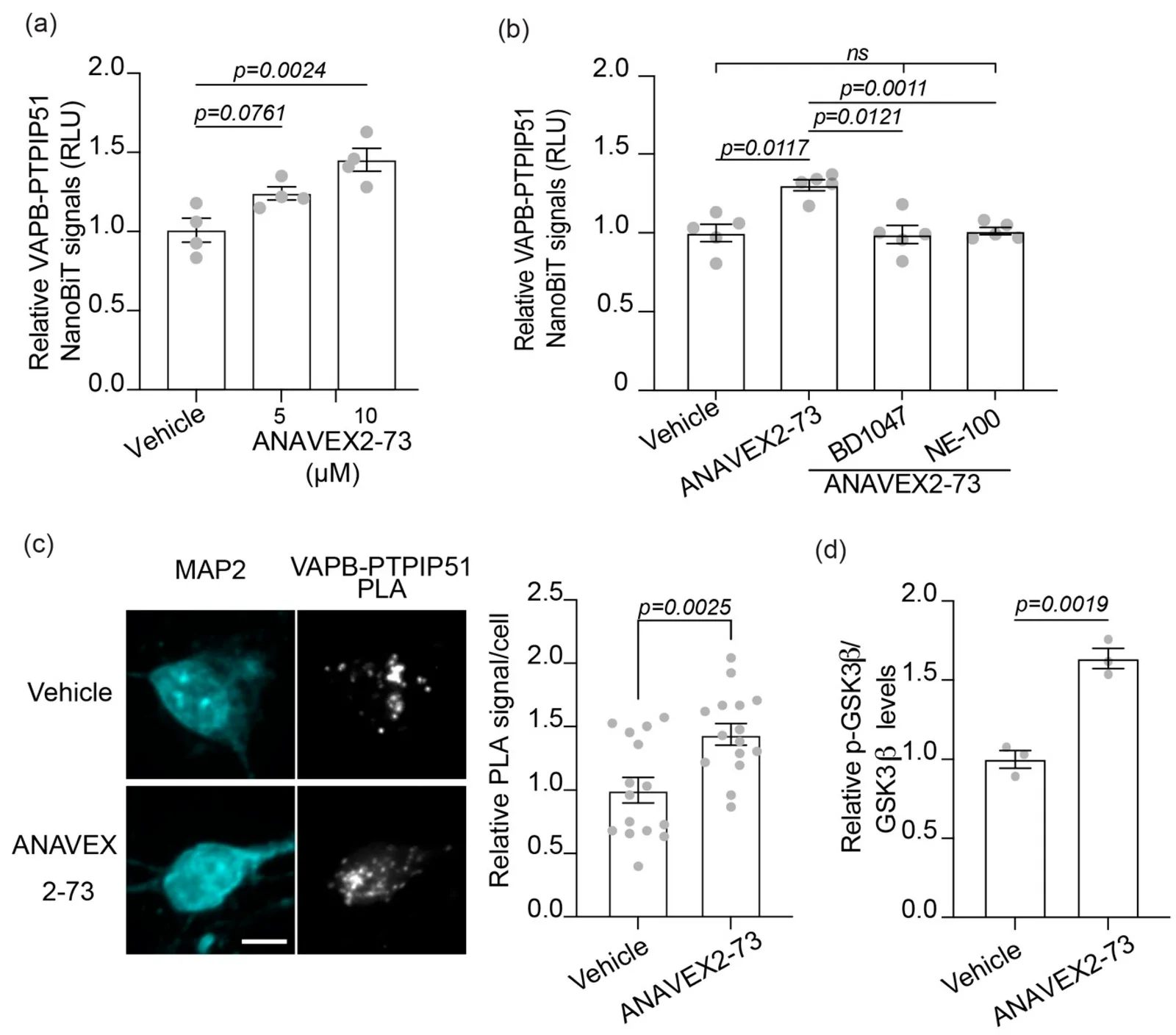
ANAVEX2-73 stimulates VAPB-PTPIP51 binding and inhibits GSK3β activity. (**a**) The effect of ANAVEX2-73 on VAPB-PTPIP51 binding was determined in SH-SY5Y cells using VAPB-PTPIP51 NanoBiT luciferase assays. Cells were co-transfected with SBiT-VAPB and PTPIP51-LBiT plasmids and treated with vehicle or 5 µM or 10 µM of ANAVEX2-73 for 1 h. Treatment with 10 µM of ANAVEX2-73 induced a significant increase in VAPB-PTPIP51 binding. Data was obtained from 4 independent experiments (N = 4 per experiment). Error bars are SEM. Statistical analysis was carried out using one-way ANOVA and Tukey’s post hoc test. *p* values are shown. F(2, 9) =11.65. (**b**) Treatment with the Sigma-1 receptor antagonists BD1047 and NE-100 blocked the stimulatory effect of ANAVEX2-73 on VAPB-PTPIP51 binding. SH-SY5Y cells were transfected with VAPB+PTPIP51 NanoBiT plasmids and then treated with 10 µM of ANAVEX2-73 and either vehicle, 10 µM of BD1047 or 10 µM of NE-100 for 1 h. Data were obtained from 5 independent experiments (N = 5 per experiment). Error bars are SEM. Statistical analysis was carried out using Welch’s one-way ANOVA and Dunnett’s T3 multiple comparisons test. *p* values are shown; ns, not significant. W(3, 8.34) = 15.8. (**c**) ANAVEX2-73 stimulated VAPB-PTPIP51 binding in PLAs in cortical neurons. Representative images are shown for neurons treated with either vehicle or 10 µM of ANAVEX2-73 for 1 h. Neuronal identity was confirmed by MAP2 immunostaining (shown in cyan). Scale bar = 5 µm. Data were obtained from 15 cells per treatment from 3 independent experiments. Error bars are SEM. Statistical analysis was performed using 2-tailed Welch’s *t*-test. *p* value is shown. t(27.14) = 3.333. (**d**) ANAVEX2-73 reduced GSK3β activity in SH-SY5Y cells. Cells were treated with vehicle or ANAVEX2-73 (10 µM for 1 h), and GSK3β activity was determined using ELISAs to quantify the levels of total and serine-9-phosphorylated GSK3β. The levels of serine-9-phosphorylated GSK3β (*p*-GSK3β) are shown after normalisation to total GSK3β levels and are displayed relative to vehicle-treated control cells. Data were obtained from 3 independent experiments. Error bars are SEM. Statistical analysis was performed using Welch’s *t*-test. *p* value is shown. t(3.901) = 7.474.

We first monitored the effect of ANAVEX2-73 on the VAPB-PTPIP51 interaction using NanoBiT assays in transfected SH-SY5Y cells. 10 µM of ANAVEX2-73 stimulated VAPB-PTPIP51 binding in these assays (Figure 5a). This concentration is in line with findings from other studies that investigated the cellular effects of stimulating the Sigma-1 receptor with ANAVEX2-73 [79]. We also confirmed that this effect of ANAVEX2-73 on VAPB-PTPIP51 binding involved Sigma-1 receptor stimulation by treating the cells with ANAVEX2-73 in combination with the Sigma-1 receptor antagonists BD1047 or NE-100. Co-treatment with BD1047 or NE-100 abolished the stimulatory effect of ANAVEX2-73 on VAPB-PTPIP51 binding (Figure 5b).

To complement these assays, we also monitored the effect of ANAVEX2-73 on endogenous VAPB-PTPIP51 binding in cortical neuron cells using PLAs. Again, 10 µM of ANAVEX2-73 stimulated VAPB-PTPIP51 binding in these assays (Figure 5c). Finally, we examined whether ANAVEX2-73 might inhibit GSK3β activity. To assess this, ELISA-based measurements were used to quantify the relative levels of serine-9-phosphorylated GSK3β in SH-SY5Y cells following treatment with either vehicle or 10 µM of ANAVEX2-73. As was the case with PRE-084, ANAVEX2-73 increased serine-9 GSK3β phosphorylation in these assays (Figure 5d). Interestingly, others have presented in vivo data which show that ANAVEX2-73 inhibits GSK3β activity [75]. Our results thus confirm these earlier findings. Thus, like PRE-084, ANAVEX2-73 stimulates VAPB-PTPIP51 binding, at least in part, by inhibition of GSK3β.

## 4. Discussion

Loss of function mutations in the Sigma-1 receptor can cause some familial forms of FTD/ALS and related neuropathies, and these mutants disrupt ER–mitochondria contacts and related IP3 receptor delivery of Ca^2+^ to mitochondria [13,14,15]. Moreover, Sigma-1 receptor agonists have beneficial effects in models of FTD/ALS [13,14,51,52,53,54]. However, the mechanisms by which such agonists exert their protective effects are not properly known.

One route involves stimulation of IP3 receptor function since the Sigma-1 receptor binds directly to IP3 receptors to facilitate mitochondrial Ca^2+^ delivery [12,13]. Another route involves stimulation of ER–mitochondria contact formation since Sigma-1 receptor agonists rescue disrupted ER–mitochondria contact formation and signalling induced by FTD/ALS insults, although the underlying mechanisms are not fully clear [13,14,80]. Sigma-1 receptor stimulation may also target the AAA ATPase domain-containing protein 3A (ATAD3A) to inhibit mitochondrial fragmentation or affect ER stress responses such as those seen in FTD/ALS [81,82]. Finally, Sigma-1 receptor agonists may provide protection against antioxidant generation in FTD/ALS via nuclear factor erythroid-2 related factor 2 signalling [67].

**Figure 6:**
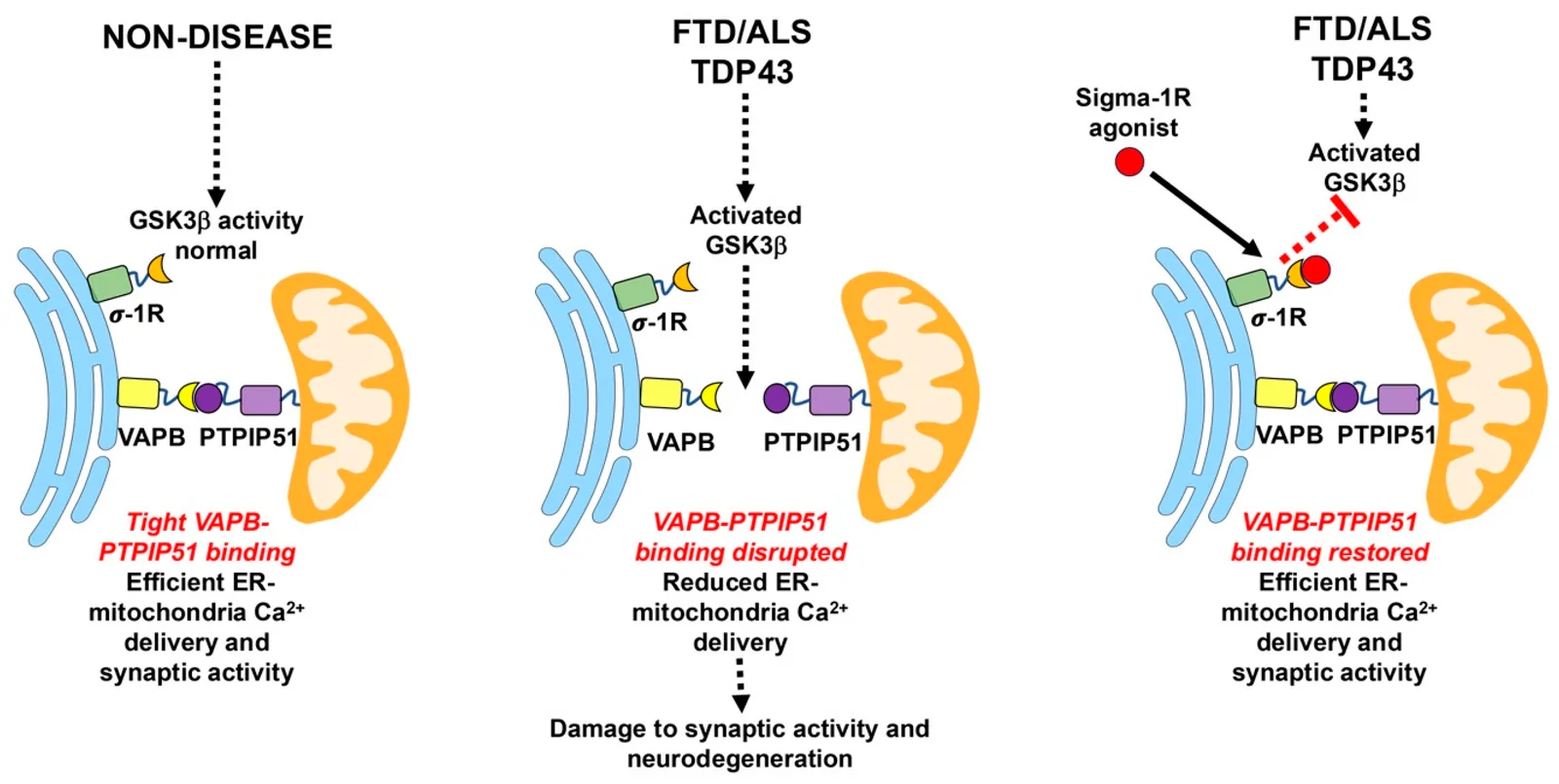
Diagram illustrating the protective effects of Sigma-1 receptor agonists on disrupted ER–mitochondria signalling in TDP43-linked FTD/ALS. Mutant TDP43 activates GSK3β to disrupt VAPB-PTPIP51 binding, ER–mitochondria Ca^2+^ delivery and synaptic function. Sigma-1 receptor agonists abrogate the effect of TDP43 on GSK3β activation to restore VAPB-PTPIP51 binding, ER–mitochondria Ca^2+^ delivery and synaptic function.

Here, we show that the Sigma-1 receptor agonist PRE-084 targets the VAPB-PTPIP51 ER–mitochondria tethering proteins. We demonstrate that PRE-084 stimulates binding of VAPB to PTPIP51 and rescues damaged VAPB-PTPIP51 binding and linked IP3 receptor delivery of Ca^2+^ to mitochondria induced by FTD/ALS mutant TDP43. We also show that these effects involve inhibition of TDP43-induced activation of GSK3β. GSK3β is a negative regulator of VAPB-PTPIP51 binding and ER–mitochondria contact formation [7,16,19,41,42,44,46,72,73]. As such, our findings provide key information on the mechanisms by which Sigma-1 receptor agonists offer protection in FTD/ALS (see Figure 6 for diagrammatic summary).

Although our study focussed on mutant TDP43, a number of other FTD/ALS-linked genetic insults have been shown to disrupt ER–mitochondria signalling. These include mutants of *FUS* (encoding fused in sarcoma), *SOD1* (encoding Cu/Zn superoxide dismutase-1), *MAPT* (encoding Tau), *C9orf72* and recessive mutations in *SIGMAR1* and *TBK1* (encoding Tank binding kinase-1) [13,14,16,42,44,46,80,83,84,85,86,87,88]. For mutant TDP43, FUS, Tau and *C9orf72*, this disruption involves reduced binding of VAPB to PTPIP51 via activation of GSK3β [16,42,43,44,45,46,47,48] (see also reviews [7,19,72,73]). Thus, the effect of PRE-084 is likely to be protective against damaged ER–mitochondria signalling caused by these other mutant genes.

The mechanisms by which Sigma-1 receptor stimulation inhibits TDP43-induced GSK3β activation are not clear. However, Akt (also known as protein kinase-B) is a favoured kinase for phosphorylating GSK3β serine-9 to inhibit its activity; Akt is regulated by phosphatidylinositol 3′ kinase (PI3 kinase) signalling [71]. Thus, Sigma-1 receptor agonists may influence PI3 kinase/Akt signalling to inhibit GSK3β activity, and there is in vivo evidence to support this mechanism for both PRE-084 and ANAVEX2-73 [75,89]. Whether these effects involve regulating further upstream signalling pathways or chaperone roles of the Sigma-1 receptor are not clear.

We also showed that ANAVEX2-73 stimulated VAPB-PTPIP51 binding via inhibition of GSK3β activity. Unlike PRE-084, ANAVEX2-73 is not a pure Sigma-1 receptor agonist [6]. However, ANAVEX2-73 has displayed promising results in clinical trials for Alzheimer’s disease, so understanding its mode(s) of action has major disease relevance [77,78]. Interestingly, the VAPB-PTPIP51 tethers are also disrupted in affected neurons in post-mortem Alzheimer’s disease brains, and this disruption occurs relatively early, which suggests that it is a driver of disease and not some end-stage epiphenomena [90]. The protective effect of ANAVEX2-73 in Alzheimer’s disease may therefore involve stimulation of VAPB-PTPIP51 binding via GSK3β inhibition. With this in mind, it would be valuable to determine whether ANAVEX2-73 also rescues TDP43-induced damage to the VAPB-PTPIP51 tethers and associated ER–mitochondria Ca^2+^ delivery in future studies.

Aside from its role in regulating VAPB-PTPIP51 binding, GSK3β is a favoured kinase for inducing pathological hyperphosphorylation of Tau in neurofibrillary tangles in Alzheimer’s disease [91,92]. Such findings have stimulated the discovery of GSK3β inhibitors as potential therapeutics for Alzheimer’s disease, although none have made it to the clinic yet. The findings that both PRE-084 and ANAVEX2-73 inhibit GSK3β suggest that Sigma-1 receptor agonists may provide an alternative route for regulating this dementia-linked kinase.

Finally, the results we report here involve experimental manipulation of cultured mammalian cells using transfection methods. To obtain further disease relevance, future studies should include analyses of how Sigma-1 receptor agonists affect the VAPB-PTPIP51 tethers in iPS cell-derived neurons from FTD/ALS patients carrying pathogenic TDP43 mutations and in vivo studies of transgenic mice expressing these mutants. Also of value would be determining whether Sigma-1 receptor agonists rescue TDP43-induced damage to synaptic activity and lipid metabolism. Synaptic loss and changes to lipids are major features of FTD/ALS, and these are regulated by the VAPB-PTPIP51 tethers [24,41,46,93,94]. Such experiments would be particularly important for ANAVEX2-73 which is in clinical trials for neurological disorders. The findings reported here provide the basis for such work.

## Data Availability

The datasets used and/or analysed during the study and the reagents are available from the corresponding author upon reasonable request.
